# Eye Tumors in Childhood as First Sign of Tumor Predisposition Syndromes: Insights from an Observational Study Conducted in Germany and Austria

**DOI:** 10.3390/cancers13081876

**Published:** 2021-04-14

**Authors:** Madlen Reschke, Eva Biewald, Leo Bronstein, Ines B. Brecht, Sabine Dittner-Moormann, Frank Driever, Martin Ebinger, Gudrun Fleischhack, Desiree Grabow, Dirk Geismar, Sophia Göricke, Maja Guberina, Claudia H. D. Le Guin, Tobias Kiefer, Christian P. Kratz, Klaus Metz, Bert Müller, Tatsiana Ryl, Marc Schlamann, Sabrina Schlüter, Stefan Schönberger, Johannes H. Schulte, Selma Sirin, Daniela Süsskind, Beate Timmermann, Saskia Ting, Werner Wackernagel, Regina Wieland, Martin Zenker, Michael Zeschnigk, Dirk Reinhardt, Angelika Eggert, Petra Ritter-Sovinz, Dietmar R. Lohmann, Norbert Bornfeld, Nikolaos Bechrakis, Petra Ketteler

**Affiliations:** 1 Department of Pediatric Hematology and Oncology, Charité-Universitätsmedizin, 13353 Berlin, Germany; madlen.reschke@charite.de (M.R.); Johannes.schulte@charite.de (J.H.S.); angelika.eggert@charite.de (A.E.); 2 Department of Ophthalmology, University Hospital Essen, University Duisburg Essen, 45122 Essen, Germany; Eva.biewald@uk-essen.de (E.B.); claudia.LeGuin@uk-essen.de (C.H.D.L.G.); tobias.kiefer@uk-essen.de (T.K.); sabrina.schlueter@uk-essen.de (S.S.); Norbert.bornfeld@uk-essen.de (N.B.); Nikolaos.bechrakis@uk-essen.de (N.B.); 3 Institute of Biostatistics and Clinical Research, University of Muenster, 48149 Münster, Germany; leo.bronstein@ukmuenster.de; 4 Department of Pediatric Hematology and Oncology, Children’s University Hospital Tübingen, 72076 Tübingen, Germany; Ines.Brecht@med.uni-tuebingen.de (I.B.B.); Martin.Ebinger@med.uni-tuebingen.de (M.E.); 5 Department of Pediatric Hematology and Oncology, University Hospital Essen, University Duisburg Essen, 45122 Essen, Germany; sabine.dittner-Moormann@uk-essen.de (S.D.-M.); Gudrun.fleischhack@uk-essen.de (G.F.); tatsiana.ryl@uk-essen.de (T.R.); stefan.schoenberger@uk-essen.de (S.S.); regina.wieland@uk-essen.de (R.W.); dirk.reinhardt@uk-essen.de (D.R.); 6 Institute of Pathology, University Hospital Essen, University Duisburg-Essen, 45122 Essen, Germany; frankoliver.driever@uk-essen.de (F.D.); Klaus.metz@uk-essen.de (K.M.); Saskia.ting@uk-essen.de (S.T.); 7 Division of Childhood Cancer Epidemiology, German Childhood Cancer Registry at Institute of Medical Biostatistics, Epidemiology and Informatics (IMBEI), University Medical Center of the Johannes Gutenberg University Mainz, 55131 Mainz, Germany; desiree.grabow@uni-mainz.de; 8 Clinic for Particle Therapy, West German Proton Therapy Centre Essen (WPE), University Hospital Essen, 45122 Essen, Germany; dirk.geismar@uk-essen.de (D.G.); Beate.timmermann@uk-essen.de (B.T.); 9 Department of Diagnostic and Interventional Radiology and Neuroradiology, University Hospital Essen, 45122 Essen, Germany; Sophia.goericke@uk-essen.de (S.G.); Selma.sirin@uk-essen.de (S.S.); 10 Department for Radiotherapy, University Hospital Essen, 45122 Essen, Germany; maja.guberina@uk-essen.de; 11 Department of Pediatric Hematology and Oncology, Hannover Medical School, 30625 Hannover, Germany; Kratz.Christian@mh-hannover.de; 12 Department of Ophthalmology, Charité-Universitätsmedizin, 13353 Berlin, Germany; bert.mueller@charite.de; 13 Department of Neuroradiology, University Hospital Köln, 50937 Köln, Germany; marc.schlamann@uk-koeln.de; 14 Department of Ophthalmology, University Hospital Tübingen, 72076 Tübingen, Germany; daniela.suesskind@med.uni-tuebingen.de; 15 German Consortium for Translational Cancer Research (DKTK), Standort Essen/Düsseldorf, 45122 Essen, Germany; Dietmar.lohmann@uk-essen.de; 16 Department of Ophthalmology, Medical University of Graz, 8036 Graz, Austria; werner.wackernagel@medunigraz.at; 17 Institute of Human Genetics, University Magdeburg, 39120 Magdeburg, Germany; martin.zenker@med.ovgu.de; 18 Institute of Human Genetics, Medical Faculty, University Duisburg-Essen, 45122 Essen, Germany; michael.zeschnigk@uni-due.de; 19 Division of Pediatric Hematology/Oncology, Department of Pediatrics and Adolescent Medicine, Medical University of Graz, 8036 Graz, Austria; petra.sovinz@klinikum-graz.at

**Keywords:** retinoblastoma, uveal melanoma, retinal astrocytoma, ciliary body medulloepithelioma, genetic counseling, DICER1 syndrome, nevus Ota, cancer predisposition syndrome

## Abstract

**Simple Summary:**

Eye tumors in children are very rare. In Europe, these eye tumors are nearly always diagnosed early and cure rates are high. However, eye tumors in childhood often occur as the first sign of a genetic tumor predisposition syndrome. This study collected data of children with malignant eye tumors diagnosed in five years in Germany and Austria to learn about the association of eye tumors in childhood with tumor predisposition syndrome. The study recruited 300 children with malignant eye tumors in childhood. In the here-presented cohort, more than 40% of eye tumors were associated with rare tumor predisposition syndromes. For this reason, all children with eye tumors and their families should receive genetic counseling for a tumor predisposition syndrome. Children with a genetic predisposition to cancer should receive a tailored surveillance, including detailed history, physical examination and, if indicated, imaging to screen for other cancers later in life.

**Abstract:**

Retinoblastoma and other eye tumors in childhood are rare diseases. Many eye tumors are the first signs of a genetic tumor predisposition syndrome and the affected children carry a higher risk of developing other cancers later in life. Clinical and genetic data of all children with eye tumors diagnosed between 2013–2018 in Germany and Austria were collected in a multicenter prospective observational study. In five years, 300 children were recruited into the study: 287 with retinoblastoma, 7 uveal melanoma, 3 ciliary body medulloepithelioma, 2 retinal astrocytoma, 1 meningioma of the optic nerve extending into the eye. Heritable retinoblastoma was diagnosed in 44% of children with retinoblastoma. One child with meningioma of the optic nerve extending into the eye was diagnosed with neurofibromatosis 2. No pathogenic constitutional variant in *DICER1* was detected in a child with medulloepithelioma while two children did not receive genetic analysis. Because of the known association with tumor predisposition syndromes, genetic counseling should be offered to all children with eye tumors. Children with a genetic predisposition to cancer should receive a tailored surveillance including detailed history, physical examinations and, if indicated, imaging to screen for other cancer. Early detection of cancers may reduce mortality.

## 1. Introduction

Malignant eye tumors are a rare disease in childhood and have been associated with tumor predisposition syndromes [1,2]. A tumor predisposition syndrome is a heritable disorder caused by a constitutional pathogenic variant in a gene in which variants confer a higher likelihood of developing certain types of neoplasia, compared with the level of risk in the general population [3]. Despite their rarity, the recognition of tumor predisposition syndromes is highly clinically relevant in directing cancer prevention strategies [4]. Genetic predisposition is an established cause of childhood cancer [2]. While some types of tumors are rarely associated with a known genetic predisposition, certain other tumor types are per se highly suggestive for the presence of an underlying tumor predisposition syndrome [5,6].

The most common malignant eye tumor in childhood is retinoblastoma. It occurs in about 1 out of 16,000–18,000 live births per year [7,8,9]. Heritable retinoblastoma is a tumor predisposition syndrome caused by a constitutional pathogenic *RB1* variant. Children with heritable retinoblastoma are at risk of developing multiple retinoblastoma in both eyes in the first years of life and other extraocular malignancies later in life, mainly soft tissue sarcoma or osteosarcoma [10,11]. They are also at risk of developing trilateral retinoblastoma, a rare primary central nervous system (CNS) tumor that histopathologically resembles retinoblastoma and occurs in the pineal or suprasellar region, usually in the first five years of life [12,13].

Other malignant intraocular tumors with onset in childhood are ciliary body medulloepithelioma, uveal melanoma and retinal astrocytoma. Ciliary body medulloepithelioma has been described in patients with DICER1 syndrome, a tumor predisposition syndrome caused by constitutional pathogenic *DICER1* variants [14]. In addition to ciliary body medulloepithelioma, individuals with DICER1 syndrome may develop pleuropulmonary blastoma, ovarian sex cord-stromal tumors, renal tumors, sarcoma and brain tumors including pineoblastoma and pituitary blastoma [15]. Uveal melanoma is a tumor with a median age of onset in the 6th decade of life that rarely occurs in children or adolescents, while it is the most common ocular tumor manifestation in adult patients [16,17,18]. Individuals with constitutional pathogenic variants in the *BAP1*-gene have a BAP1-Tumor predisposition syndrome (BAP1-TPDS), which is associated with an increased risk of uveal melanoma, mesothelioma, cutaneous melanoma and clear cell renal carcinoma [19,20]. Retinal astrocytoma is a rare eye tumor arising from retinal glial cells in early childhood that occur in patients with tuberous sclerosis (TSC) (57% of retinal astrocytomas) or patients with neurofibromatosis type I (14% of children with retinal astrocytoma) while 29% of children with retinal astrocytoma do not carry a known underlying tumor predisposition syndrome [21,22,23,24]. A benign retinal tumor is the retinal hemangioblastoma with an incidence of 1:40,000, which is approximately half the incidence of retinoblastoma and is commonly, but not always, associated with the von Hippel Lindau Syndrome [25].

The 5-year overall survival of children with retinoblastoma and most other pediatric eye tumors is >95% in high-income countries [26,27]. However, long-term sequelae are common and often a burden for survivors. Reported side effects include compromised vision aesthetic deficits after surgery or irradiation, platinum-induced ototoxicity and psychosocial late effects [28,29,30]. Children with heritable tumor predisposition syndrome carry the risk of developing further second primary malignancies (SPM) related to high mortality [31]. Therapeutic interventions may even increase this risk for SPM [32]. Especially in retinoblastoma, the choice of treatment modality depends on tumor stage at diagnosis [33]. While children with smaller intraocular eye tumors are increasingly treated with a wide variety of eye-preserving therapies, advanced tumors require enucleation and, in some patients, additional risk-stratified, adjuvant therapy with chemo- or radiotherapy. The major challenge is to tailor treatment for each individual patient in order to preserve life, globe and vision while reducing late sequelae. Genetic counseling and molecular genetic analysis for children with retinoblastoma and other eye tumors may identify tumor predisposition syndromes and enable participation on cancer surveillance programs and testing of other family members at risk.

Routine genetic counseling and molecular genetic analysis for tumor predisposition is well-established for children with retinoblastoma, but it is less recognized in children with other rare eye tumors. Here, prospective data from a binational, multicenter, observational study are analyzed to describe the incidence and clinical presentation of malignant eye tumors with onset in childhood and the association with tumor predisposition syndromes.

## 2. Materials and Methods

### 2.1. Patient Cohort

Since November 2013, data from all children diagnosed in Germany and Austria with either malignant eye tumors or benign eye tumors with potential local progression threatening the eye sight are collected in a multicenter observational study on pediatric eye cancer (RB-Registry, www.clinicaltrials.gov: DRKS00005423 accessed on 26 February 2021) after informed consent. Patients with benign tumors such as retinal hemangioblastoma were not included in the RB-registry. The here-presented study analyzes data of children enrolled within the first five years of recruitment (4 November 2013 to 3 November 2018) with a follow-up until the 19 November 2020. RB-registry was conducted in compliance with the Declaration of Helsinki and ethic approval was obtained from all local ethics committees of participating centers (leading ethic committee University Duisburg Essen: no 13-5405-BO from 22 August 2013). Inclusion criteria for registration were new diagnosis of pediatric eye tumors, aged below 18 years, no preceding tumor-specific therapy and permanent residence in Germany or Austria. Reference evaluations for ophthalmology, pediatric oncology, radiology, neuroradiology, genetics, cytology and histopathology were provided within RB-registry to improve comparability of diagnostic results between participating centers. Data were documented in the online database MARVIN©, xclinical, Germany.

Heritable retinoblastoma was defined as diagnosis of bilateral or trilateral retinoblastoma or familial retinoblastoma (clinical criteria) or as evidence of pathogenic variant in blood DNA (either mosaic or heterozygous (genetic criteria)). All other children were classified as non-heritable retinoblastoma or subdivided into two groups with either non-heritable retinoblastoma confirmed by genetic analysis or unilateral retinoblastoma without genetic analysis.

### 2.2. Genetic Counselling and Molecular Genetic Analysis

Association with a defined tumor predisposition syndrome has been described for each eye tumor with onset in childhood included in this study. All children with clinically suspected genetic tumor predisposition and their families or legal guardians, were offered genetic counseling and after obtaining informed consent, targeted molecular genetic analysis of a specific gene depending on the tumor entity. For children with retinoblastoma, DNA from fresh-frozen tumor tissue, if available, and blood DNA was examined to identify the pathogenic *RB1* variants as reported previously [34,35,36,37]. This included one or more of the following methods: analysis of allele loss in tumors, cytogenetic analysis, denaturing high performance liquid chromatography, exon-by-exon sequencing, multiplex ligation-dependent probe amplification, methylation-sensitive PCR, quantitative fluorescent multiplex PCR, quantitative real-time PCR, and single strand conformation polymorphism. Diagnosis of mutational mosaicism was based on the finding of a skewed signal ratio of the variant to the normal allele relative to the ratio obtained from heterozygous DNA. Children with ciliary medulloepithelioma were offered genetic analysis of the *DICER1* gene, which includes targeted exon-by-exon sequencing, multiplex ligation-dependent probe amplification (SALSA MLPA Probemix P482 *DICER1*, MRC Holland). In children with uveal melanoma, chromosome 3 status was determined in tumor DNA as described previously [38]. Molecular tumor type characterization was performed by Sanger sequencing of Exons 4 and 5 of both *GNAQ* and *GNA11* genes [39]. The child with meningioma of the optic nerve received targeted screening of the *NF2* gene.

### 2.3. Statistical Analysis

Descriptive analyses were carried out computing quantiles, absolute numbers and percentages. Event rates were estimated via the Kaplan–Meier method. Group comparisons between categorical variables were based on the chi-squared test, and between continuous variables on the Mann–Whitney-U test. The logrank-test was used to compare groups with respect to their survival distributions. All tests were two-sided. Follow-up time was defined from the date of diagnosis of eye tumor until the last date with documented clinical information in the RB-registry. Statistical analyses aimed to provide exploratory, not confirmatory, results, and no adjustment for multiple testing was performed. *p*-values ≤ 0.05 were considered statistically relevant. All statistical computations were done using R 4.0.3 (R Core Team (2020) R: A language and environment for statistical computing. R Foundation for Statistical Computing, Vienna, Austria) and IBM SPSS^®®^ Statistics 27 (IBM Corporation, Somers, NY, USA).

## 3. Results

### 3.1. Retinoblastoma Was the Most Common Malignant Eye Tumor in Childhood

**Figure 1:**
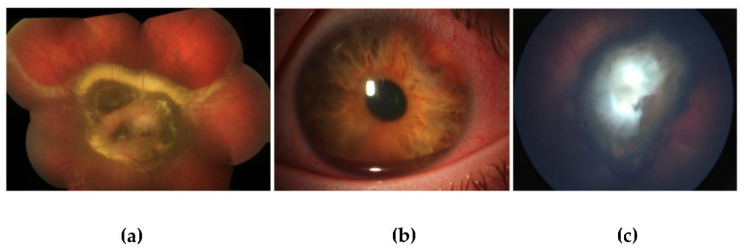
Fundoscopy images of patients with rare eye tumors with onset in childhood. (**a**) Circumpapillary, size-progressive choroidal tumor with extensive exudation. Transretinal biopsy and histopathologic workup revealed a WHO grade 1 pilocystic astrocytoma. (**b**) Nasal superiorly located ciliary body medulloepithelioma with an accompanying hyphemia, treated with a ruthenium plaque brachytherapy. (**c**) The fundoscopy image shows an optic nerve meningioma associated with *NF2*. The central mass with extensive epiretinal membranes made the optic nerve demarcation impossible.

In 5 years, 300 children were diagnosed with an eye tumor with onset in childhood in Germany and Austria (Table 1). Patients were recruited in 6 centers (5 centers in Germany, 1 center in Austria). The most common eye tumor was retinoblastoma (287 RB, 95.7%), resulting in 57.4 children diagnosed in Germany and Austria together each year. The reported number of births during this time was in the mean 845,081 per year (Germany 759,898 births/year [40] and Austria 85,183 births/year [41]), which leads to an incidence of 1 in 14,723 births. The second most common eye tumor in childhood was uveal melanoma in 7 patients (2.3%). In one of these patients, the uveal melanoma was localized at the iris, in one patient it was localized in the ciliary body infiltrating the iris and one child presented with a malignant melanoma of the orbit extending into the uvea in association with a nevus of Ota. Other registered children with eye tumors were 3 children with ciliary body medulloepitheliomas (1%), 2 with retinal astrocytomas (0.7%) and one child with meningioma of the optic nerve that extended into the eye (0.3%). The median time of documented follow-up in the RB-registry database was 2.1 years (range 0.0–6.8 years). Fundoscopic appearance of some of these rare eye tumors is shown in Figure 1.

### 3.2. The Age at Diagnosis Differed among the Different Eye Tumor Entities

**Figure 2:**
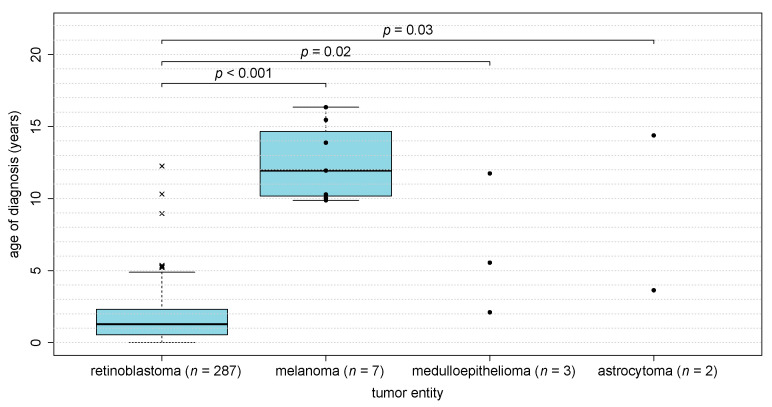
Age at diagnosis of pediatric eye tumors in childhood. Age at diagnosis differs between types of pediatric eye cancer. Boxplot “whiskers” extend to the most extreme data point not farther away from the box than 1.5 times the box height. Data points outside that range are represented by ‘x’. Filled circles represent individual data points (for groups with a small number of patients). Filled circles represent individual data points (for groups with a small number of patients).

No significant gender predilection was observed for any of the tumor entities (Table 1). Age at diagnosis differed clearly between different types of pediatric eye tumors (Table 1, Figure 2). Children with retinoblastoma (*n* = 287) had a median age of 1.3 years (range 0.0 months–12.3 years) while children with melanoma (*n* = 7) were significantly older with a median age of 11.9 years (9.9–16.3 years) (*p*_Mann-Whitney-U-Test_ < 0.001). Ciliary body medulloepithelioma and retinal astrocytoma were diagnosed at a median age of 5.5 years (2.1–11.8 years) and 9.0 years (3.6–14.4 years), respectively.

### 3.3. Eye Tumors in Childhood Are often the First Sign of a Tumor Predisposition Syndrome

Genetic analysis was completed and documented in 235 patients with retinoblastoma (81.9% of all children with retinoblastoma). Of these 235 children, 37.0% carried a heterozygous variant (87 patients), 5.1% a somatic mosaicism (12 patients) and 57.9% showed no oncogenic variant in blood DNA (136 patients). Table 2 summarizes the type of *RB1* variants diagnosed. Genetic analysis of tumor or blood DNA in children with other eye tumors was performed and documented in 6 of 13 children with other eye tumors (46.2% of all children with eye tumor other than retinoblastoma). Two of the three patients with ciliary body medulloepithelioma were treated with enucleation and one with eye-preserving therapy. One of the children with ciliary body medulloepithelioma received genetic counseling and no constitutional variant in *DICER1* was detected in blood DNA. Tumor DNA was not available because of enucleation in another center. No genetic counseling or molecular genetic analysis for somatic or constitutional variants in *DICER1* were reported for the other two patients with ciliary body medulloepithelioma. One of these two was treated with enucleation and one with eye-preserving treatment. The two children with retinal astrocytoma presented with unifocal astrocytoma and no genetic counseling or molecular analysis for TSC or NF1 was reported in the RB-Registry. One child with astrocytoma was treated with enucleation and for one child the information on therapy was missing. The two patients with iris and ciliary body melanoma and one patient with uveal melanoma had no cytogenetic analysis of tumor DNA because no tumor material was available. The remaining 4 patients with uveal melanoma received cytogenetic analysis of melanoma tissue and in 3 of 4 patients tumor DNA showed a disomy of chromosome 3. For the class of uveal melanomas with a disomy of chromosome 3, unlike tumors with monosomy 3, no tumor predisposition syndrome has been described so far and, for this reason, genetic counseling and analysis of constitutional DNA for BAP1 TPDS was not indicated. Genetic analysis of the tumor DNA in the fourth patient showed alterations on chromosome 3 that did not allow a clear assignment to the class of either monosomy 3 or disomy 3 tumors. This patient developed a systemic relapse of uveal melanoma and died of the disease. The patient with meningioma of the optic nerve extending into the eye was diagnosed with a pathogenic variant in the *NF2* gene, resulting in the clinical diagnosis of neurofibromatosis type 2 (NF2).

### 3.4. The Overall Survival of Children with Eye Tumors Is High

The 2-year overall survival and 5-year overall survival for all children with retinoblastoma registered in RB-registry was 99.5% (95% Confidence Interval (CI): 96.9–99.9%) and 96.0% (95% CI: 86.5–98.9%). The overall survival did not differ significantly between children with non-heritable and heritable retinoblastoma (5-years OS: non-heritable: 99.0% (95% CI: 94.3–99.8%), heritable 94.6% (95% CI: 80.2–98.6%) (*p*_log rank_ = 0.84). All children with retinal astrocytoma and ciliary body medulloepithelioma were alive at the last follow-up. One of the patients with uveal melanoma deceased 3.7 years after initial diagnosis. All four deceased patients (3 with retinoblastoma and 1 with uveal melanoma) died of metastatic spread of the tumor. Two of the three children with trilateral retinoblastoma deceased. One of these children deceased 3.6 years after simultaneous diagnosis of intraocular and suprasellar disease and one child 1.1 year after diagnosis of pineal retinoblastoma and 3.9 years after diagnosis of intraocular retinoblastoma. The fourth child died 1 year after diagnosis of an advanced intraocular retinoblastoma with extensive postlaminar optic nerve infiltration but without infiltration of the resection margin.

### 3.5. Trilateral Retinoblastoma Is a Rare Manifestation of Heritable Retinoblastoma

A trilateral retinoblastoma was diagnosed in 1.1% of all patients with retinoblastoma (3 of all 287 patients with retinoblastoma) and 2.3% of all patients with heritable retinoblastoma (3 of 129 patients). Two children with trilateral retinoblastoma carried a heterozygous constitutional *RB1* variant, one was not tested. Two of the three trilateral retinoblastoma were located in the suprasellar region and one in the pineal gland. One patient presented with the intracranial tumor 2.6 years prior, one simultaneously with and one 2.9 years after diagnosis of the intraocular retinoblastoma.

### 3.6. Clinical Presentation of Heritable and Non-Heritable Retinoblastoma Is Distinct

**Figure 3:**
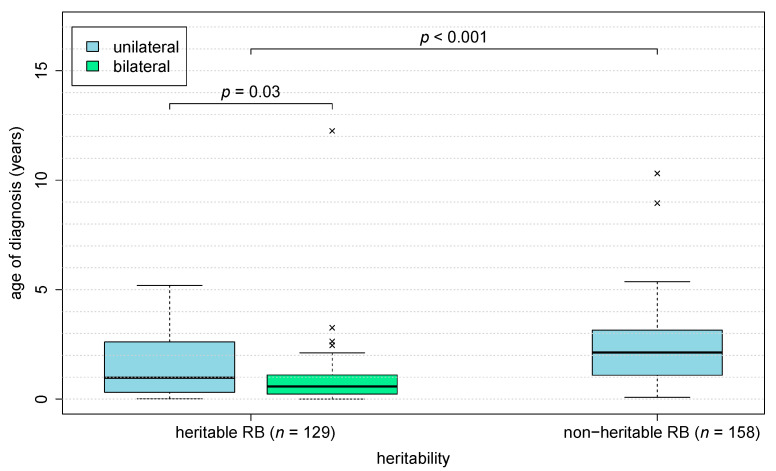
Age of diagnosis of retinoblastoma correlates with heritability. Children with heritable retinoblastoma (*n* = 129) were diagnosed earlier than patients with non-heritable retinoblastoma (*n* = 158) (p_Mann-Whitney-U-Test_ < 0.001). The four patients without data on laterality are not depicted in the plot. Boxplot “whiskers” extend to the most extreme data point not farther away from the box than 1.5 times the box height. Data points outside that range are represented by ‘x’.

**Figure 4:**
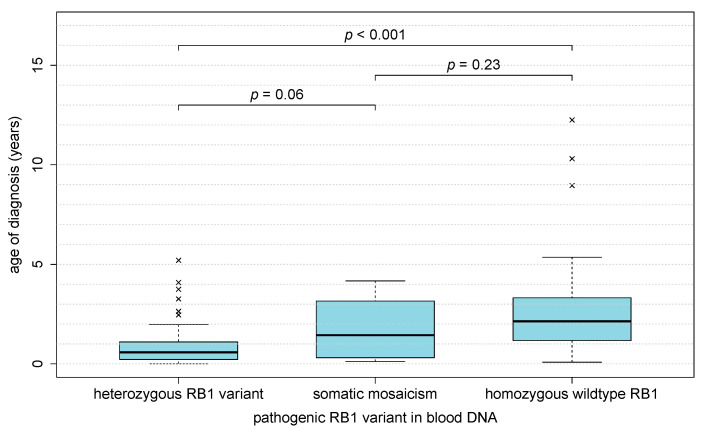
Age of diagnosis of retinoblastoma in children heterozygous for a *RB1* variant and with somatic mosaicism. Children with a heterozygous *RB1* variant showed a non-significant trend towards a lower age of diagnosis than those with somatic mosaicism (*p*_Mann-Whitney-U-Test_ = 0.06). Age of diagnosis of 52 children without genetic analysis are not shown here. Boxplot “whiskers” extend to the most extreme data point not fartheraway from the box than 1.5 times the box height. Data points outside that range are represented by ‘x’.

Heritable retinoblastoma occurred as a unilateral retinoblastoma in 21.7% (28 children) and as a bilateral retinoblastoma in 76.7% (99 children). All 156 patients with non-heritable retinoblastoma and with data on laterality developed unilateral retinoblastoma (98.7%). Data on laterality was missing in two patients with heritable retinoblastoma (1.6% of all heritable retinoblastoma) and two patients with non-heritable retinoblastoma (1.3% of all non-heritable retinoblastoma). As a result, 15.2% (28 of 184 children) of all children with unilateral retinoblastoma were diagnosed with heritable retinoblastoma. Eight patients developed metachronous bilateral retinoblastoma, defined as development of retinoblastoma in the contralateral eye more than 30 days after initial diagnosis (8.1% of all bilateral RB and 4.2% of all initially unilateral RB). Six children (5.1% of 117 patients with heritable retinoblastoma) showed a 13q deletion syndrome. One third of patients with 13q deletion syndrome developed unilateral retinoblastoma (2 unilateral, 4 bilateral). Children with heritable retinoblastoma (*n* = 129) were diagnosed at a younger age (median: 0.65 years, interquartile range: 1.0 years) than patients with non-heritable retinoblastoma (*n* = 158, median: 2.1 years, interquartile range: 2.1 years; *p*_Mann-Whitney-U-Test_ < 0.001) (Figure 3). Among children with heritable retinoblastoma, children heterozygous for a pathogenic *RB1* variant (median: 0.6 years, Interquartile range: 0.9 years) showed a non-significant trend for a younger age at diagnosis than children with somatic mosaicism (median 1.4 years, Interquartile range: 2.9, *p*_Mann-Whitney-U-Test_ = 0.06) (Figure 4).

## 4. Discussion

Eye tumors with onset in childhood are rare and all eye tumor entities included in this registry have a known association with genetic predisposition for childhood cancers. Genetic predisposition is best described for retinoblastoma and 81.9% of all children with retinoblastoma in this study received genetic counseling and molecular genetic analysis while only 45% of the children with other eye tumor entities received genetic analysis of tumor DNA or blood DNA. In total, more than 40% of children presenting with eye tumors had an underlying tumor predisposition syndrome.

During the first 5 years, the registry recruited 60 children per year with newly diagnosed eye tumors from specialized eye tumor centers in Germany and Austria. Retinoblastoma was by far the most common eye cancer with onset in childhood diagnosed in 57 children per year in Germany and Austria together. The incidence of 1 patient with retinoblastoma in 14,723 births was slightly higher than the estimated incidence of 1 in 15,000–18,000 from previous studies [9]. An even higher increase in reported incidence was observed in a recent study on incidence of retinoblastoma in 40 European countries that corresponded with a higher proportion of familial retinoblastoma [42]. Other eye tumors with onset in childhood, like ciliary body medulloepithelioma, retinal astrocytoma or uveal melanoma, were extremely rare. However, there is a potential recruitment bias because benign eye tumors such as hamartomas (retinal astrocytoma) are not always referred to oncologists and are therefore not always included in the registry. In addition, the name RB-registry is misleading to support inclusion of all eye tumors with onset in childhood.

Nearly 45% of children diagnosed with retinoblastoma were diagnosed with a heritable tumor predisposition syndrome. In line with previous reports, clinical presentation of retinoblastoma differed between heritable and non-heritable retinoblastoma in regards to age at diagnosis and laterality [26]. The majority of patients with heritable retinoblastoma were diagnosed with bilateral disease. Incidence of metachronous retinoblastoma (2.8% of 287 patients) was rare and in line with 1.5–3.1% in other reports [43,44]. Age at diagnosis was significantly younger in patients with heritable retinoblastoma than with non-heritable retinoblastoma, but the range was broad [45]. Especially children with somatic mosaicism of *RB1* variant presented with a higher age at diagnosis in the group of children with heritable retinoblastoma. Despite these differences in clinical presentation of heritable and non-heritable retinoblastoma regarding age at diagnosis and laterality, a tumor predisposition syndrome cannot be excluded clinically. All patients with bilateral, trilateral or familial retinoblastoma have to be considered as carriers of a heritable tumor predisposition syndrome, even if no molecular genetic analysis was performed or in the absence of detection of the oncogenic *RB1* variant in blood DNA. The majority of patients with sporadic unilateral retinoblastoma has non-heritable retinoblastoma, but only molecular genetic analysis can identify the minority among these that carry a constitutional *RB1* variant and heritable retinoblastoma [46]. Knowledge of the tumor predisposition syndrome and of the risk for metachronous bilateral retinoblastoma enables the clinicians to adjust the treatment strategy and follow-up intensity and to screen family members at risk, especially young siblings. For this reason, genetic counseling is particularly important for patients with unilateral retinoblastoma and their families.

Not only retinoblastoma but also other types of rare eye tumors have been described as first signs of heritable tumor predisposition syndromes. Ciliary body medulloepithelioma can be a manifestation of the tumor predisposition syndrome DICER1 Syndrome [47]. No constitutional variant in *DICER1* has been reported in patients with medulloepithelioma in the registry, but in the past, children with ciliary body medulloepithelioma did not routinely receive genetic counseling and molecular genetic analysis for variants in the *DICER1* gene. Additionally, in 30% (4 of 13 patients) of all children with an eye tumor other than retinoblastoma, the tumor-carrying eye was not enucleated and no tumor tissue was available for diagnostics. Uveal melanoma may be the first sign of BAP1 TPDS and until today children are not routinely assessed for constitutional pathogenic *BAP1* variants in Germany. However, melanoma tissue is routinely examined for monosomy 3 and, if proven, for somatic *BAP1* variants. In the here-presented cohort of 7 children with uveal melanoma, four patients received cytogenetic analysis of tumor DNA and no patient showed monosomy 3. Children with monosomy 3 in uveal melanoma tissue should be offered genetic counseling and molecular genetic analysis of constitutional DNA. Retinal astrocytoma are benign tumors of the retina that occur sporadic or as first signs of genetic syndromes such as TSC or NF1 [21,22,23]. Children with retinal astrocytoma need careful pediatric examination for other signs of genetic syndrome and may benefit from genetic counseling. Meningioma of the optic nerve has been described as a very rare presentation of neurofibromatosis 2 (NF2) in infancy and initial presentation of optic nerve meningioma as an eye tumor is more unusual [48]. The affected patient in the registry carried a de novo pathogenic variant in the *NF2* gene [48]. All of these tumor predisposition syndromes, associated with eye tumors in childhood, are heritable in an autosomal dominant manner. For this reason, the diagnosis of the tumor predisposition syndrome may have implications for other family members.

In the here-presented study, the 5-year overall survival rate of children with retinoblastoma was 96.0%. This survival rate for retinoblastoma is similar to reports from other European and North American countries [3,33,34]. Out of 287 children with retinoblastoma, two children deceased because of trilateral retinoblastoma and one because of advanced disease with deep infiltration of the optic nerve at diagnosis. Only children with heritable retinoblastoma are at risk of developing an intraocular and intracranial retinoblastoma in childhood (trilateral retinoblastoma). The incidence in the here-presented study was 2.4% of patients with heritable retinoblastoma, which is slightly lower than the 5% incidence described in other studies [13,49]. The low number of patients and the short time of follow-up for some patients in this study may explain the difference. Even in children with intraocular retinoblastoma, the risk of metastasis and fatal outcome should never be underestimated, but the death of 2 in 3 patients with trilateral retinoblastoma underlines the poor prognosis of intracranial retinoblastoma. The benefit of screening examinations for intracranial retinoblastoma has not been shown [12] but early diagnosis of genetic predisposition is necessary to council children at risk and their families. All children with medulloepithelioma and retinal astrocytoma in this series survived, but one of seven patients with uveal melanoma died of metastatic disease 3.7 years after diagnosis. The current number of patients does not allow in-depth analysis, emphasizing the need to continue the recruitment in the registry to learn about these very rare eye tumors and their genetic background.

All of the tumor entities included in this study are mentioned as criteria for referral to genetic counseling in the recommendations for screening of children for tumor predisposition syndromes [50,51]. Molecular genetic analysis of paired tumor and blood samples facilitates the diagnosis and the exclusion of a heritable tumor predisposition syndrome. Many eye tumors are treated conservatively and the lack of tumor tissue may complicate genetic counseling. Liquid biopsy has been suggested as a potential technique to detect the oncogenic variants in the tumor tissue to then exclude constitutional variants in the future [52]. Children with rare pediatric tumor predisposition syndromes are at risk of developing other primary malignancies later in life and tailored guidelines and recommendation for life-long surveillance have been established for most tumor predisposition syndromes [4]. The aim of surveillance protocols is to detect other cancers early and thereby reduce mortality from cancers later in life [1]. The benefit of a life-long biochemical and imaging surveillance on survival has been shown for children with Li-Fraumeni Syndrome, a tumor predisposition syndrome caused by constitutional *TP53* variants [53]. Children with heritable retinoblastoma require regular ophthalmological examinations in the first years of life to detect further retinoblastoma foci and treatment for retinoblastoma needs to be tailored to avoid radiotherapy [10]. Lifelong surveillance for individuals with heritable retinoblastoma include a detailed history and physical examinations for signs of other cancers, especially sarcoma and melanoma, and irregular findings should prompt immediate imaging [4,54,55]. Imaging-based surveillance for DICER1-associated cancers varies depending on the age of the individual, as the risk for cancers is highest in early childhood and decreases in adulthood [15]. Surveillance for children with NF1 includes regular clinical assessment by a pediatrician and an ophthalmologist, while children with NF2 are recommended to attend at least an annual brain MRI and clinical examination with auditory assessment [56,57]. Recommendations for surveillance are updated regularly and it is crucial that individuals with cancer predisposition syndrome are connected to a specialized clinic for surveillance.

## 5. Conclusions

Retinoblastoma and other eye tumors with onset in childhood are associated with rare tumor predisposition syndromes in childhood. Retinoblastoma is the most frequently diagnosed eye tumor in children and most children with retinoblastoma in Germany and Austria routinely receive genetic counseling. However, other malignant eye tumor entities are extremely rare in childhood and the association with tumor predisposition syndrome is less recognized. For this reason, genetic counseling and, if available, paired analysis of tumor and blood samples should be considered in all patients with eye tumors in childhood.

## Figures and Tables

**Table 1:** Clinical characteristics of children with eye cancer in childhood other than retinoblastoma.

Clinical Characteristics		Retinoblastoma	Uveal Melanoma	Ciliary Body Medulloepithelioma	Retinal Astrocytoma	Meningioma Optic Nerve with Intraocular Extension
all		287	7	3	2	1
gender	female	138	4	1	1	0
	male	149	3	2	1	1
age at diagnosis of eye tumor (years)	median	1.3	11.9	5.5	9.0	0.28
	range	0.0–12.3	9.9–16.3	2.1–11.8	3.6–14.4	
status	alive	284	6	3	2	1
	deceased	3	1	0	0	0
laterality	unilateral	184	7	3	2	1
	bilateral	99				
	no data	4				
Known associated cancer predisposition genes		*RB1*	*BAP1*	*DICER1*	*TSC1* *TSC2* *NF1*	*NF2*
Pathogenetic variant in cancer predisposition gene	yes	99	0	0	0	1
	no evidence	136	3	1	0	0
	not tested or inconclusive	52	4	0	0	0
	Number of affected eyes	386	7	3	2	1

**Table 2:** Genetic and clinical characteristics of children with retinoblastoma.

Genetic and Clinical Criteria		Number of Patients (in %)
**All**		**287**
heritability of retinoblastoma	heritable RB	129 (44.9)
(clinical and/or genetic criteria)	non-heritable RB with genetic analysis	130 (45.3)
	unilateral without genetic analysis	28 (9.8)
genetic analysis of *RB1*	constitutional *RB1* variant	87 (30.3)
	somatic mosaic *RB1* variant in blood	12 (4.2)
	no *RB1* variant detected in blood	136 (47.4)
	no data	52 (18.1)
type of heterozygous constitutional variant	nonsense or frameshift variant	36 (41.4)
	splice-site variant	29 (33.3)
	missense or in-frame	8 (9.2)
	large deletion	14 (16.1)
family history	isolated	246 (85.7)
	familial	33 (11.5)
	data missing	8 (2.8)
number of affected eyes		386
ICRB Group	ICRB A	37 (9.6)
	ICRB B	87 (22.5)
	ICRB C	21 (5.4)
	ICRB D	70 (18.1)
	ICRB E	147 (38.1)
	no data	24 (6.2)

ICRB, international classification of retinoblastoma; n/a, not applicable; RB, retinoblastoma.

## Data Availability

Aggregated data are available on personal request to the authors.
